# Development of Food Group Tree-Based Analysis and Its Association with Non-Alcoholic Fatty Liver Disease (NAFLD) and Co-Morbidities in a South Indian Population: A Large Case-Control Study

**DOI:** 10.3390/nu14142808

**Published:** 2022-07-08

**Authors:** Amrita Vijay, Amina Al-Awadi, Jane Chalmers, Leena Balakumaran, Jane I. Grove, Ana M. Valdes, Moira A. Taylor, Kotacherry T. Shenoy, Guruprasad P. Aithal

**Affiliations:** 1 Inflammation, Injury and Recovery Sciences, School of Medicine, University of Nottingham, Nottingham NG7 2UH, UK; amrita.vijay@nottingham.ac.uk (A.V.); ana.valdes@nottingham.ac.uk (A.M.V.); 2 National Institute for Health Research (NIHR) Nottingham Biomedical Research Centre, Nottingham University Hospitals NHS Trust and the University of Nottingham, Nottingham NG7 2UH, UK; amina.alawadi@nottingham.ac.uk (A.A.-A.); j.chalmers@nhs.net (J.C.); jane.grove@nottingham.ac.uk (J.I.G.); 3 Nottingham Digestive Diseases Centre, Translational Medical Sciences, School of Medicine, University of Nottingham, Nottingham NG7 2UH, UK; 4 Population Health Research Institute (PHRI), Trivandrum, Kerala 695011, India; leenakb@yahoo.com (L.B.); dr.ktshenoy@gmail.com (K.T.S.); 5 School of Life Sciences, Faculty of Medicine and Health Sciences, University of Nottingham, Nottingham NG7 2UH, UK; moira.taylor@nottingham.ac.uk

**Keywords:** NAFLD, South Asians, dietary factors, case-control, co-morbidities, food groups

## Abstract

Background: Non-alcoholic fatty liver disease (NAFLD) is a global problem growing in parallel to the epidemics of obesity and diabetes, with South Asians being particularly susceptible. Nutrition and behaviour are important modifiers of the disease; however, studies to date have only described dietary patterns and nutrients associated with susceptibility to NAFLD. Methods: This cross-sectional case-control study included 993 NAFLD patients and 973 healthy controls from Trivandrum (India). Dietary data was collected using a locally validated food frequency questionnaire. A tree-based classification categorised 2165 ingredients into three levels (food groups, sub-types, and cooking methods) and intakes were associated with clinical outcomes. Results: NAFLD patients had significantly higher consumption of refined rice, animal fat, red meat, refined sugar, and fried foods, and had lower consumption of vegetables, pulses, nuts, seeds, and milk compared to controls. The consumption of red meat, animal fat, nuts, and refined rice was positively associated with NAFLD diagnosis and the presence of fibrosis, whereas consumption of leafy vegetables, fruits, and dried pulses was negatively associated. Fried food consumption was positively associated with NAFLD, whilst boiled food consumption had a negative association. Increased consumption of animal fats was associated with diabetes, hypertension, and cardiovascular outcomes among those with NAFLD, whereas consumption of wholegrain rice was negatively associated with these clinical-related outcomes. Conclusions: The tree-based approach provides the first comprehensive method of classifying food intakes to enable the identification of specific dietary factors associated with NAFLD and related clinical outcomes. This could inform culturally sensitive dietary guidelines to reduce risk of NAFLD development and/or its progression.

## 1. Introduction

A diet in which energy intake exceeds expenditure over a prolonged period of time results in deposition of excessive body fat—namely obesity—subsequent insulin resistance [1], altered lipid metabolism, and non-alcoholic fatty liver disease (NAFLD) [2,3,4]. People of Indian (South Asian) ethnicity are at increased risk of diabetes and other metabolic complications at a lower body mass index (BMI) than Caucasians [5,6]. This has been compounded by Westernisation of Asia-Pacific culture [7], resulting in higher rates of NAFLD in this ethnic group [8,9].

The role of diet in the development of NAFLD has been investigated previously. However, the focus of these studies has primarily been on differences in total energy intake or the role of specific macro- or micronutrient components of diet and NAFLD risk [3,10,11,12,13,14,15]. Such studies, whilst contributing to our understanding of the role of diet in NAFLD, are limited to the specific nutrients with sufficiently valid and comprehensive analytical data. Food composition tables do not include many components believed to impact health, such as phenolics, and foods may include as yet uncharacterised active components. Furthermore, by considering nutrients in isolation from the diverse, complex, and structurally varied entities in which they are consumed, we diminish the potential to gain new insights into the relation between diet and NAFLD of direct and practical relevance to a local area, where diet is shaped by local culture, economics, and availability of foods [16].

There are some studies within the literature that have looked at the links between specific dietary patterns and presence of NAFLD [17,18]. These studies appear to show that the “Simple diet” from East Asia composed principally of vegetables and grains, or the Mediterranean diet, which is low in saturated fat and high in unsaturated fatty acids, confer the least risk of NAFLD [19,20]. Evidence to support such approaches are limited [21]. Neither of these dietary patterns have direct relevance within the Indian population. India has a rich and highly varied cuisine, comprising foods that are created from complex recipes that differ geographically in relation to social identity, religion, local agricultural practices, and availability of diverse foods [22]. This makes analysis of Indian nutritional geometry in relation to metabolic disease and NAFLD difficult, particularly due to the limitations of current dietary data collection processes. There have been advances in dietary data collection and analysis techniques, including the New Interactive Nutrition Assistant—Diet in India Study of Health method (NINA-DISH), which combines detailed dietary recall strategies, generating data that can be used to examine links between 12 broad food groups, expanded into 20 more specific food groups, with disease [23]. More recently, the concept of food group hierarchy has also been used to examine the impact of diet, microbiome, and health [24]. The concept of dietary hierarchy, from nutrients to foods, meals, and subsequent diet, is an attractive way to analyse all components of nutrition in relation to disease and could in turn inform culturally sensitive dietary guidelines.

A unique dietary dataset collected in the Trivandrum NAFLD cohort captures both individual dietary intake and detailed composition of local recipes, facilitating novel analysis by food group and comparison to clinical outcomes. Utilising these data, this study provides a methodological approach for developing a food group, tree-based analysis across three levels: 1. Main food groups (for individual items and composite dishes), 2. Refined sub-classifications at the ingredient level, and 3. Cooking method applied. Correlations will be explored between dietary intake and NAFLD outcomes amongst the NAFLD cohort in comparison with healthy controls. This approach will facilitate the unprecedented exploration of the role of dietary indicators associated with NAFLD in a particular cohort and describe a method that can be translated to different populations, for whom comparable dietary data are available.

## 2. Materials and Methods

### 2.1. Study Design

The current study is a cross-sectional nested case-control study. Trivandrum (Thiruvananthapuram) is the southern-most district in the state of Kerala, located on the south-west coast of India. At the time of the Indian census in 2011, it had a population of 3.3 million divided into urban and rural domiciles (urban 54%, rural 46%) [25]. The Trivandrum NAFLD cohort was originally designed and set up in 2013 to examine the interaction between genetics and lifestyles factors that result in increased risk of NAFLD within this population. The cohort also gives an accurate estimation of population prevalence of NAFLD and enables analysis of impact of different variables on NAFLD risk. The Trivandrum NAFLD cohort was created between February 2013 and July 2016 through population-based sampling of all inhabitants over the age of 25 years; the development of the cohort and the details of case-control definition have been described previously [26]. The enrolment of study participants was through unweighted multi-stage cluster sampling of the whole population [26]. Dietary data were collected at the time of recruitment, through house-to-house survey by local social workers, and participants attended local study camps to undergo ultrasound to identify those with NAFLD; those with liver fat on ultrasound were classified as cases and those without as controls [26]. Within the final cohort (*n* = 2158), the NAFLD prevalence was 49.8%. A proportion of those with NAFLD (*n* = 688) underwent transient elastography (TE) via Fibroscan to identify those with evidence of significant fibrosis (liver stiffness ≥ 8.4 kPa) [27].

### 2.2. Ethical Approval

Ethical approval for the study was granted by the Sree Gokulam Medical College and Research Foundation, Venjaramoodu, Trivandrum ethics committee. This study and all relevant documentation received approval from the University of Nottingham Faculty of Medicine and Health Sciences Research Ethics Committee (REC: 26/299/05/2017, 14/06/17) and the Nottingham University Hospitals NHS Trust Research and Development department.

### 2.3. Clinical Outcomes

Clinical data were collected at local study camps. BMI was calculated from height (m), measured using a stadiometer, and weight (kg) using standing scales. Asian cut-offs for BMI categories (kg/m^2^) were used-(<18.5 underweight, 18.5–23 normal, 23–27.5 overweight, and ≥27.5 obese). Presence of diabetes (history of diabetes and/or fasting glucose > 126 mg/dL), hypertension (history of hypertension, antihypertensives, and/or systolic BP > 130, diastolic BP > 85), dyslipidaemia (lipid-lowering therapy, triglycerides > 150 mg/dL, and/or HDL < 50 g/dL for women, or HDL < 40 g/dL for men), and cardiovascular disease (history of myocardial infarction or stroke) were identified through documentation of past medical history, measurement of blood pressure, and results of biochemical blood tests. Presence of significant fibrosis on TE (liver stiffness ≥ 8.4 kPa) was used as a surrogate for significant liver disease as an outcome.

### 2.4. Dietary Data

Dietary data for this study were collected by trained nutritionists using the Population Health and Research Institute (PHRI) food frequency questionnaires (FFQs), which were validated against three-day food records, and self-reported 24-h recalls [25]. The PHRI-FFQ consists of 361 recipes and 28 single food items (Indian foods) in 13 sections and is designed to classify participants according to the average daily intake level of energy, nutrients, and food items/recipes during the past one year. Participants were instructed to record their intake in multiples of a reference serve size, which was described using a household measure for which the researchers knew the weight in grams. Having estimated the quantity consumed when an item was consumed, participants then selected from ten responses how frequently each item was consumed during the last year, ranging from highest to lowest intake (e.g., from >6, 4–6, 2–3, 1 per day; 5–6, 2–4, 1 per week; once per month, occasionally/seasonally, to never). Full dietary data were available for 2047 participants.

A database was provided by PHRI, which gave a breakdown of individual ingredients for each recipe that was present in the reference serve size for that recipe. The number of ingredients per recipe ranged from 2 to 20.

### 2.5. Deconstruction of the Recipes into Ingredients, and Food-Groups Tree Development

**Figure 1:**
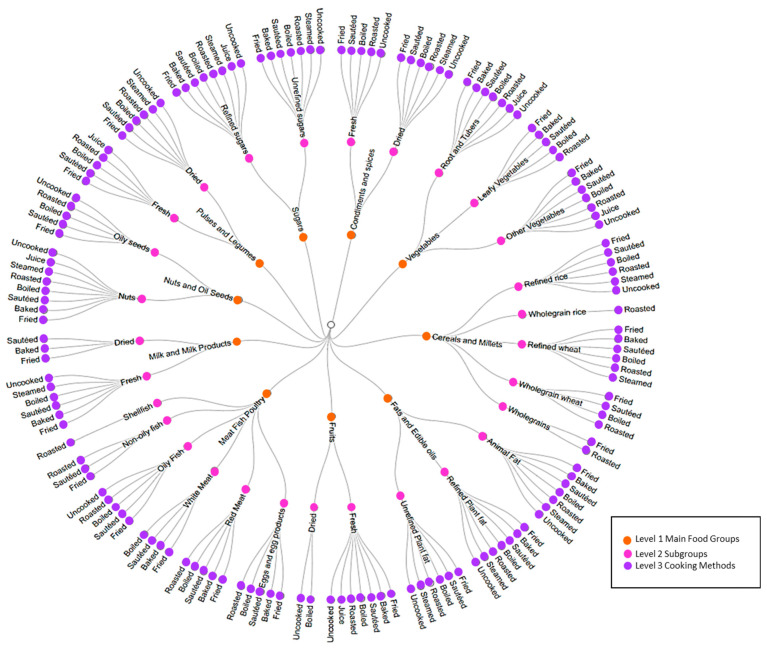
Representation of the food group levels based on the proposed food tree structure. Level 1 of the tree (inner circle) represents the 10 main food groups. Level 2 of the tree (middle circle) represents the 23 sub classifications related to each main food group in level 1. Level 3 (outer circle) represents the 8 cooking/processing methods related to sub classifications in level 2.

The 361 recipes listed in the PHRI-FFQ were expanded into 2165 individual ingredients. Each individual ingredient, and any of the 28 individual food items listed separately on the FFQ that had not been listed in a recipe, were categorised at 3 levels using a tree-based classification ranging from the main food groups to cooking method as shown in Figure 1.

The first level of the food-groups tree hierarchy in the present study was developed based on the food groups of the Indian Food Composition Tables (IFCT) database [28]. It consisted of ten main food groups, which were taken from published food composition tables (for example, ‘cereals and millets’). The second level consisted of 29 sub-classifications providing a more precise description of the food (for example ‘cereals and millets’ included the sub classification ‘wholegrain rice’). Selection of sub-classifications was undertaken as an iterative process, informed by the known list of individual ingredients created from the FFQ, and the authors’ knowledge of associations between food characteristics and health (for example, vegetables were categorised into root, leafy, and other veg to reflect differences in starch and vitamin content). Level three was intended to classify food items according to how they had been processed/cooked in the recipe from which they had been derived, again with reference to potential functional impact (e.g., steamed as opposed to boiled impacting water soluble vitamin content, and fruit-juiced compared with ‘uncooked/unprocessed’ having a disrupted structural matrix, impacting glycaemic response). Eight cooking/processing methods were identified. The circular dendrogram chart (Figure 1) shows the food groups in each level and the 3-level tree combinations.

### 2.6. Ingredient’s Intake Calculations

A single unit for each ‘reference local food serve’ was described to participants in terms of a household measure, and they described how many of the reference units they would have on an occasion and indicated how frequently they would consume this amount of food. The reference local food serves were then converted into weights (grams) by the PHRI group using their local knowledge of the weight of the household measures used as the single unit for the reference food serve.

The intake (gram per day; g/day) of each recipe/food item for each participant was first calculated using the following formula: reference serve size x number of reference servings on an occasion x frequency of consumption factor. The frequency of consumption factor used to estimate the intake was created by selecting the appropriate conversion factor to adjust the reported intake to the amount consumed per day. Individual ingredient intakes of each participant were calculated using the cooked weight of participant intake but raw weights for the recipe as follows:

Ingredient Intake per participant (g/day) =

$$\frac{Amount\;of\;Recipe\;\;consumed\;by\;particpant\;}{Total\;weight\;of\;recipe\;in\;the\;reference\;serve\;size\;\;}\times weight\;of\;ingredient\;in\;reference\;serves\;size$$

Participants’ energy and macronutrient intakes were calculated by the multiplication of ingredient intake per participant (g/day) by the nutrient composition (g/100 g) calculated using the IFCT tables [28]. DietSoft software, an Indian-based program that has been developed for the analysis of Indian food generated from IFCT was used [28]. Uncooked food composition was used for all ingredients.

### 2.7. Identification of Outliers

Energy intake (EI) to basal metabolic rate (BMR) [EI:BMR] ratio was calculated for each participant. BMR was estimated using sex-specific prediction equations, which include age and body weight [29]. EI:BMR bottom cut-off values were calculated as = Mean − (2 × SD; standard deviation) and EI:BMR top cut-off values were calculated as = Mean + (2 × SD) [30]. Cut-off values for the control cohort were (≥0.8352 or ≤3.0508), and cut-off values for the NAFLD cohort were (≥0.7426 or ≤2.8774). A total of 53 participants from the control cohort (19 under-reporters and 34 over-reporters) and 28 participants from the NAFLD cohort (6 under-reporters and 22 over-reporters) were excluded. This resulted in the final analytical dataset of 973 participants in the control cohort and 993 participants in the NAFLD cohort as outlined in the participants flow chart [Supplementary Figure S1].

### 2.8. Statistical Analysis

Baseline data from the cohort were presented as follows: Categorical data were presented as numbers (percentage; %) and continuous data were presented as mean (±SD). Intakes were adjusted by dividing absolute mean intakes per person per day by their respective body weights and were represented as the daily average adjusted intakes per kg body weight. Daily intakes of each food group (g/day) were adjusted for body weight and comparison between NAFLD and control groups was performed using an unpaired *t*-test. The associations of the unadjusted daily intakes of each food group with NAFLD status and clinical outcomes were analysed through logistic regressions adjusted for age, gender and weight or BMI. Linear regressions were performed to assess the association of food group intakes with advanced liver fibrosis (TE scores) amongst the NAFLD group. All analyses were performed using IBM/SPSS Statistics (version 25.0, IBM, Armonk, NY, USA) and R version number 3.6.1. *p* < 0.05 was considered statistically significant.

## 3. Results

Demographics and clinical characteristics are described in Table 1. Participants with NAFLD were older, had a higher BMI, and had higher prevalence of components of the metabolic syndrome. Rates of cardiovascular disease outcomes were similar between cases and controls (*p* = 0.06). In those with NAFLD, there was evidence of significant fibrosis in 22.82% (*n* = 157).

### 3.1. Association of Food Intakes and Cooking Methods with NAFLD

The differences in weight-adjusted mean intakes of the different food groups between cases and controls are outlined in Table 2. The adjusted intakes (g/kg/day) of cereals and millets; fats and edible oils; meat, fish, and poultry; and sugars were significantly higher in the NAFLD group compared with controls. The NAFLD group had significantly higher intakes of refined rice (5.48 g/kg/day vs. 4.61 g/kg/day), red meat (0.11 g/kg/day vs. 0.08 g/kg/day), and refined sugars (0.59 g/kg/day vs. 0.48 g/kg/day) as compared with controls (*p ≤* 0.05), whereas the intakes of vegetables, pulses and legumes, nuts and oil seeds, and milk and milk products were lower in the NAFLD group compared to controls.

**Figure 2:**
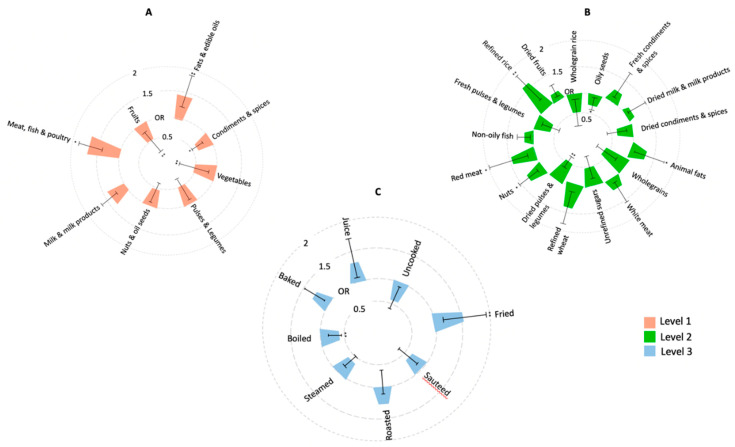
CIRCOS plot showing the association of individual food group levels (**A**-Level 1; **B**-Level 2; **C**-Level 3) with NAFLD status. Bars represent Odds ratio’s (ranging from 0.5–2) and 95% CIs from logistic regressions of unadjusted intakes, adjusted for age, gender and weight. (* *p* < 0.05; ** *p* < 0.001).

The associations between food groups and NAFLD is summarised in Figure 2. Food groups such as meat, fish, and poultry and fats and edible oils and were associated significantly with a greater risk of susceptibility to NAFLD, respectively. (OR [95% CI]) = 1.53 [1.23–1.74]), *p* = 0.039; (OR [95% CI] 1.40 [1.31–1.82]), *p* < 0.001) in the NAFLD group. Whereas intakes of vegetables (OR [95% CI]) = 0.48 [0.22–0.61], *p* < 0.001), fruits (OR [95% CI] = 0.46 [0.27–0.67] *p* < 0.001), and condiments and spices (OR [95% CI] = 0.64 [0.5–0.71], *p* = 0.042) food groups significantly reduced the susceptibility. On evaluating the sub classifications in level 2, we found positive associations with the intakes of refined rice (OR [95% CI]) = 1.53 [1.31–1.73], *p* < 0.001), animal fat (OR [95% CI]) = 1.39 [1.20–1.56], *p* = 0.029), red meat (OR [95% CI]) = 1.48 [1.21–1.82]), *p* = 0.031), and nuts (OR [95% CI]) = 1.21 [1.12–1.52], *p* = 0.042) in the NAFLD group. However, the strongest negative association was found with the intake of dried pulses and legumes (OR [95% CI] = 0.43 [0.21–0.61], *p* = 0.001).

We further investigated if different cooking methods were associated with NAFLD. Based on the unpaired *t*-test, we found that the NAFLD group consumed significantly higher fried and roasted foods and significantly lower boiled, steamed, and uncooked/unprocessed foods compared to the control group (g/kg/day). Furthermore, we found that the consumption of fried foods was positively associated with NAFLD status (OR [95% CI] = 1.49 [1.22–1.78], *p* = 0.016), whereas a significant negative association was found with boiled food consumption and NAFLD (OR [95% CI] = 0.43 [0.20–0.48], *p* = 0.001).

### 3.2. Association of Food Groups Intakes with Presence of Significant Liver Fibrosis

NAFLD individuals with evidence of significant fibrosis had higher intakes of fats and edible oils (0.45 g/kg/day vs. 0.21 g/kg/day; *p* < 0.05), red meat (0.09 g/kg/day vs. 0.05 g/kg/day; *p* < 0.05), and fried foods (0.22 g/kg/day vs. 0.14 g/kg/day; *p* < 0.05) compared to those without (Table 3). However, a negative association was seen between the consumption of leafy vegetables and presence of significant fibrosis (Beta (SE) = −0.081 (0.032), *p* = 0.029).

**Figure 3:**
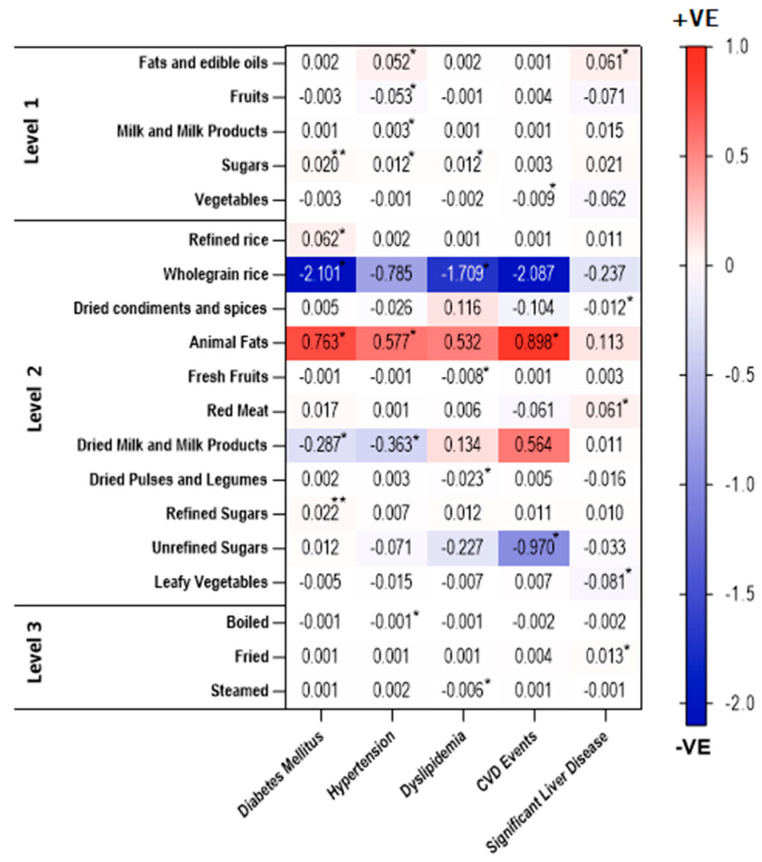
Heatmap showing positive and negative associations of the intakes of significant food groups with clinical outcomes amongst individuals with NAFLD. The axis represents the range of positive (red) and negative (blue) associations. *p* values adjusted for age, gender, and BMI (* *p* < 0.05; ** *p* < 0.001).

In addition to the associations with presence of significant liver fibrosis, we analysed the association of intakes of three-tree food group levels with diabetes mellitus, hypertension, dyslipidaemia, and cardiovascular events (Figure 3, Supplementary Table S1). We observed positive associations between intakes of sugars, refined sugars (*p* < 0.001), refined rice, and animal fat (*p* < 0.05) with Type 2 Diabetes. In addition, wholegrain rice as well as dried milk and milk products had negative associations with diabetes mellitus (*p* < 0.05). Positive associations were found between hypertension and intakes of fats and edible oils, sugars, milk and milk products, and animal fats, whereas intakes of fruits and dried milk and milk products were negatively associated with hypertension (*p* < 0.05). We found that sugar intake was positively associated with dyslipidaemia. However, the intake of wholegrain rice, fresh fruits, dried pulses, and legumes had negative associations with dyslipidaemia. Like the findings of diabetes mellitus and hypertension, animal fat intake was positively associated with CVD events. Remarkably, we observed negative associations between vegetables and unrefined sugars intakes and CVD events (*p* < 0.05).

Regarding cooking methods, negative associations were observed between boiled food intake and hypertension, and between steamed food intake and dyslipidaemia.

## 4. Discussion

This study is the first of its kind, providing a methodological approach for food group analysis for links between diet and disease. It includes ingredient-level analysis of dietary intake, taken from the detailed breakdown of local complex recipes within a large population-based cohort, and has enabled analysis of the associations of different food group levels with presence of NAFLD and other clinical-related outcomes.

In line with other studies [31,32,33], our data have shown that within this population, those with NAFLD consume more cereals (as refined rice), fats and edible oils (as animal fat), meat (as red meat), and sugars (as refined sugar) than controls. They consume fewer vegetables, pulses and legumes, nuts, and dairy products. Through logistic regression analysis, consumption of meat (animal fat and red meat specifically) was strongly associated with susceptibility to NAFLD, and with more significant liver fibrosis—a finding that is mirrored in the recent meta-analysis of NAFLD and diet (OR = 1.12, CI 1.04–1.21, *p* = 0.002) [34]. Refined dietary carbohydrates consumption has been linked with insulin resistance and increased hepatic de novo lipogenesis [35,36,37]. The high saturated fat content in red meat has been shown to induce hepatic fat accumulation and insulin resistance via lipid oxidation reduction and lipid synthesis acceleration [36,38,39]. Nuts are nutrients-dense food known to have potential protective effects on NAFLD in the general population, such nutrients are fibre, antioxidants, and unsaturated fatty acids (such as MUFAs and PUFAs) [40]. However, the effects of nut intake among NAFLD patients in existing literature still controversial [41,42,43]. Within this population, we found a positive association between nuts consumption and NAFLD, which is the converse to the meta-analysis data (OR = 0.84, CI 0.73–0.97, *p* = 0.014) [34]. A recent case-control study has found that NAFLD patients who consumed more nuts (17.75–38.09 g/day) had higher NAFLD risk (OR, 3.03; CI, 1.03–8.90; *p* = 0.046) as compared with those with lower consumption (0.55–12.3 g/day). Authors have attributed this effect to the high daily energy intake among this population [44]. In addition, our analysis showed that nuts (coconuts and cashew nut) consumed mostly as roasted and fried (e.g., Achappam, Halwa). It has been shown that cooking methods influence the composition of health-related nutrients in nuts [45,46], which might be implicated to our findings. Similar to other studies [47], consumption of fried food was also positively associated with NAFLD and presence of significant fibrosis, whilst boiled food consumption had a negative association. Thermal oxidation of fats during the frying process results in the production of several toxic compounds such as radical species that have been shown to induce biomolecule damage [48] and contributes to denaturation of liver enzymes [49]. Among our study population, unrefined plant fats (coconut oil) were consumed mainly fried. Exposing such oils to high heat alters the configuration of unsaturated fatty acid bonds to be saturated (from *Cis* to *trans* isomers), which have been corelated with increased serum levels of liver enzymes and LDL cholesterol [50].

Increased consumption of fats and red meat was strongly associated with significant liver fibrosis, while consumption of leafy vegetables was negatively associated with significant liver fibrosis. Our results are in agreement with the findings of Soleimani et al. (2019) [51], which showed positive association between high consumption of red meat and fats with hepatic fibrosis risk. They also concluded that adherence to healthy dietary patterns (characterized by high vegetables intake) was associated with lower odds of fibrosis (OR: 0.26; 95% CI: 0.10–0.49, *p* = 0.011) [51]. Leafy vegetables are also shown to have potential protective effects in reducing liver fat accumulation due to their high content of nitrate compounds in NAFLD but not in patients with advanced hepatic fibrosis [52,53].

Our findings of food group intakes and related-clinical outcomes are consistent with the literature [54,55,56,57]. Increased intakes of refined sugars, refined rice, and animals had adverse effects on related clinical outcomes, whilst unrefined sugar (Jaggery), wholegrain rice, fruit, milk, and vegetable intakes had protective effects on these outcomes. Effects of animal fat consumption (from ghee) are possibly due to the high content of saturated fats and cholesterol found in ghee [58], which has been linked with insulin resistance [36,38,59]. In contrast, high content of fibre, vitamins, minerals, and phytochemicals found in vegetables, fruits, and wholegrains have been suggested to improve insulin sensitivity and glucose metabolism [55,60].

This study utilises data from a large, deeply phenotyped, population-based cohort, and provides the first comprehensive method to refine food intakes into ingredients and assess associations with NAFLD. Few epidemiologic studies have adopted the concept of food group intake analysis in relation to the risk of NAFLD development and/or progression [31,32]. However, the selection of food groups varies among studies and has been tailored based on the existing information in relation to a particular population. Furthermore, the studies have used categories that include heterogenous items (e.g., baked products) that could cover very different items, whereas we have overcome such issue by breaking down food recipes to ingredients. However, we may have circumvented the impact of ingredients interaction by deconstructing to the ingredient level. The tree-based approach enabled the identification of specific dietary indicators associated with NAFLD and co-morbidities, which could serve as culturally sensitive dietary guidelines to reduce risk of NAFLD development and/or progression.

Our study has some limitations. First, the PHRI-FFQ used in the dietary assessment had no sections to measure salt, soft drinks, and alcohol intakes, although people with excess alcohol consumption (men and women consuming >21 unit and >14 units, respectively) were excluded. Second, physical activity levels and biomarkers associations were not analysed. Furthermore, the relationships between clinical outcomes could confound our interpretation that diet and the clinical outcomes are associated. Third, we have used uncooked ingredients weights from standard local recipes, and this could affect the nutrient content. For example, the nutrients content of the ingredient ‘raw chicken’ in uncooked curry will be different to that in the cooked curry. This accuracy could be improved by getting each household to provide the recipes and the proportion of the total recipe consumed by the patient. We have excluded under- and over-reporters of energy intake; however, we were unable to distinguish between under-reporting and deliberate energy restriction. The current tree-based approach is a descriptive analysis utilizing the various food group levels and their association with presence or absence of NAFLD. Therefore, the current study does not explore the interactions between food groups and cooking methods, and this would be a separate body of work to be considered in the future. Finally, we note that the cardiovascular disease of 2.52% in NAFLD group and 2.16% in the control group are in the lower end of the range observed in India (1.6–7.4% in rural and 1% to 13.2% in urban population [61]. Potential explanations of this observation may be that our cohort included people aged 25 years or more with a mean age of 48 years in NAFLD group and 46 years in control group [26]. Cross-sectional design, exclusion of risk factors such as alcohol intake over 21 units, and zero prevalence of smoking among women in this cohort would have reduced the risk further.

## 5. Conclusions

The tree-based classification provides a practical approach to identify the influence of diet on NAFLD, beyond dietary patterns and nutrients. This enables us to identify dietary risk factors of NAFLD. The study findings expand our knowledge in understanding the interplay between diet and disease, which could be translated into meaningful dietary recommendations with potential public health benefits. The current approach can be translated and applied to different populations, where food data is readily available.

## Figures and Tables

**Table 1:** Baseline characteristics of the cohort.

	NAFLD (*n* = 993)	Control (*n* = 973)	*p*-Value
Age, years, mean (SD)	48.16 (10.73) *	45.90 (12.32)	<0.001
Male gender (*n*, %)	453 (45.62) *	316 (32.48)	<0.001
Weight (kg, mean SD)	68.36 (11.57) *	60.78 (11.12)	<0.001
BMI (kg/m^2^, mean SD) - Underweight (*n*, %) - Normal weight (*n*, %) - Overweight (*n*, %) - Obese (*n*, %)	26.87 (4.13) * 3 (0.30) 158 (15.91) 187 (18.83) 645 (64.95)	24.33 (4.07) 61 (6.27) 310 (31.86) 206 (21.17) 396 (40.70)	<0.001 <0.001 <0.001 0.195 <0.001
Significant liver fibrosis (*n*, %) *n* = 688	157 (22.82)	N/A	
Diabetes (*n*, %)	334 (33.64) *	180 (18.50)	<0.001
Hypertension (*n*, %)	396 (39.88) *	288 (29.60)	<0.001
Dyslipidaemia (*n*, %)	695 (69.99) *	520 (53.44)	<0.001
Cardiovascular disease (*n*, %)	25 (2.52)	21 (2.16)	0.598

* *p* < 0.05 for NAFLD vs. Control group.

**Table 2:** Comparison of mean intakes of individual food groups and their associations with control and NAFLD groups.

	CONTROL (*n* = 973) Adjusted Mean Intake g/kg/day (±SD)	NAFLD (*n* = 993) Adjusted Mean Intake g/kg/day (±SD)
**FOOD GROUP LEVEL-1**		
Cereals and Millets	5.34 (±1.68)	6.16 * (±1.98)
Condiments and Spices	0.37 (±0.18)	0.33 * (±0.17)
Fats and Edible Oils	0.32 (±0.14)	1.48 * (±1.13)
Fruits	1.15 (±1.07)	1.13 (±1.12)
Meat, Fish, and Poultry	1.96 (±1.37)	2.20 * (±1.48)
Milk and Milk Products	2.55 (±1.56)	2.33 * (±1.46)
Nuts and Oil Seeds	1.24 (±0.50)	1.21 * (±0.32)
Pulses and Legumes	0.74 (±0.33)	0.70 * (±0.36)
Sugars	0.5 (±0.35)	0.62 * (±0.40)
Vegetables	3.48 (±1.66)	3.24 * (±1.51)
**FOOD GROUP LEVEL-2**		
Refined Rice	4.61 (±1.68)	5.48 * (±1.97)
Refined Wheat	0.14 (±0.10)	0.13 * (±0.11)
Wholegrain Rice	0.002 (±0.00)	0.001 (±0.00)
Wholegrain Wheat	0.56 (±0.64)	0.5 (±0.59)
Wholegrains	0.02 (±0.03)	0.021 (±0.04)
Dried Condiments and Spices	0.32 (±0.17)	0.29 * (±0.15)
Fresh Condiments and Spices	0.051 (±0.02)	0.04 * (±0.02)
Animal Fats	0.008 (±0.01)	0.01 (±0.01)
Refined Plant Fat	0.22 (±0.12)	0.21 (±0.11)
Unrefined Plant Fat	0.1 (±0.056)	0.09 * (±0.05)
Dried Fruits	0.02 (±0.026)	0.01 * (±0.02)
Fresh Fruits	1.13 (±1.06)	1.11 (±1.12)
Eggs and Egg Products	0.15 (±0.13)	0.13 * (±0.13)
Non-Oily Fish	0.03 (±0.039)	0.02 * (±0.04)
Oily Fish	1.72 (±1.38)	1.52 * (±0.23)
Shellfish	0.006 (±0.054)	0.008 (±1.24)
Red Meat	0.08 (±0.12)	0.11 * (±0.011)
White Meat	0.19 (±0.23)	0.21 (±0.20)
Dried Milk and Milk Products	0.009 (±0.015)	0.006 * (±0.01)
Fresh Milk and Milk Products	2.54 (±1.56)	2.33 * (±1.45)
Nuts	1.22 (±0.49)	1.10 * (±0.42)
Oily Seeds	0.01 (±0.008)	0.02 (±0.011)
Dried Pulses and Legumes	0.58 (±0.33)	0.56 (±0.30)
Fresh Pulses and Legumes	0.16 (±0.15)	0.14 (±0.12)
Refined Sugars	0.48 (±0.34)	0.59 * (±0.39)
Unrefined Sugars	0.02 (±0.027)	0.02 (±0.027)
Leafy Vegetables	0.27 (±0.25)	0.24 (±0.21)
Other Vegetables	1.83 (±0.93)	1.70 * (±0.87)
Roots and Tubers	1.38 (±0.75)	1.29 * (±0.68)
**FOOD GROUP LEVEL-3**		
Baked	0.08 (±0.12)	0.07 (±0.10)
Boiled	8.38 (±2.64)	7.26 * (±2.37)
Fried	0.77 (±0.50)	0.79 * (±0.53)
Roasted	1.25 (±0.67)	1.35 * (±0.73)
Sauteed	5.04 (±2.18)	4.59 (±1.97)
Steamed	1.36 (±0.75)	1.27 * (±0.68)
Juice	0.09 (±0.11)	0.1 (±0.11)
Uncooked	1.78 (±1.53)	1.67 * (±1.47)

* *p* < 0.05 compared to controls.

**Table 3:** Comparison of mean intakes of individual food groups and their associations with degree of fibrosis based on liver stiffness measurements.

	Absence of Fibrosis (≤8.4 kPa) (N = 543) (g/kg/day) ^	Presence of Fibrosis (>8.5 Pa) N = 161 (g/kg/day) ^	Beta Coefficient	S.E.	*p*-Value
	*t*-Test		Regression Analysis		
**FOOD GROUP LEVEL-1**					
Cereals and Millets	1.27	1.29	0.012	0.008	0.652
Condiments and Spices	0.16	0.11	−0.086	0.011	0.092
Fats and Edible Oils	0.21	0.45 *	0.061	0.031	0.021 *
Fruits	1.21	0.20	−0.071	0.022	0.782
Meat, Fish, and Poultry	1.10	1.17	0.051	0.041	0.075
Milk and Milk Products	1.21	1.15	0.015	0.011	0.148
Nuts and Oil Seeds	0.07	0.06	−0.007	0.003	0.614
Pulses and Legumes	0.10	0.08	−0.015	0.006	0.425
Sugars	0.26	0.29	0.021	0.016	0.512
Vegetables	1.21	1.18	−0.062	0.046	0.091
**FOOD GROUP LEVEL-2**					
Refined Rice	2.18	2.20	0.011	0.007	0.081
Refined Wheat	0.03	0.02	0.032	0.028	0.318
Wholegrain Rice	0.001	0.001	−0.237	0.204	0.317
Wholegrain Wheat	0.22	0.21	−0.021	0.011	0.421
Wholegrains	0.001	0.001	−0.041	0.021	0.211
Dried Condiments and Spices	0.11	0.19	−0.012	0.021	0.011 *
Fresh Condiments and Spices	0.01	0.02	−0.207	0.116	0.076
Animal Fats	0.002	0.006	0.113	0.108	0.137
Refined Plant Fat	0.12	0.11	0.001	0.021	0.719
Unrefined Plant Fat	0.04	0.02	0.023	0.014	0.241
Dried Fruits	0.005	0.003	0.002	0.013	0.712
Fresh Fruits	0.08	0.05	0.003	0.001	0.112
Eggs and Egg Products	0.08	0.10	0.003	0.006	0.182
Non-Oily Fish	0.001	0.002	0.004	0.011	0.641
Oily Fish	1.06	1.05	0.006	0.004	0.251
Shellfish	0.002	0.001	−0.021	0.011	0.237
Red Meat	0.05	0.09 *	0.061	0.033	0.031 *
White Meat	0.09	0.10	0.005	0.003	0.214
Dried Milk and Milk Products	0.001	0.002	0.011	0.018	0.341
Fresh Milk and Milk Products	0.02	0.03	0.012	0.011	0.719
Nuts	0.07	0.06	0.003	0.001	0.733
Oily Seeds	0.004	0.003	−0.512	0.319	0.202
Dried Pulses and Legumes	0.20	0.18	−0.016	0.008	0.111
Fresh Pulses and Legumes	0.08	0.04	−0.010	0.004	0.261
Refined Sugars	0.19	0.21	0.010	0.008	0.211
Unrefined Sugars	0.002	0.001	−0.033	0.026	0.191
Leafy Vegetables	0.11	0.10 *	−0.081	0.032	0.029 *
Other Vegetables	1.11	1.13	−0.016	0.013	0.082
Roots and Tubers	1.05	1.08	−0.008	0.002	0.282
**FOOD GROUP LEVEL-3**					
Baked	0.01	0.02	0.002	0.001	0.818
Boiled	1.18	1.21	−0.002	0.001	0.111
Fried	0.14	0.22 *	0.013	0.002	0.031 *
Roasted	1.15	1.18	0.001	0.001	0.457
Sautéed	1.19	1.18	0.001	0.003	0.614
Steamed	1.11	1.08	−0.001	0.001	0.365
Juice	0.02	0.04	0.006	0.007	0.44
Uncooked	1.18	1.16	−0.002	0.001	0.912

Unpaired t-test for differences in mean intakes for presence vs. absence of fibrosis among NAFLD group * *p* < 0.05. ^ adjusted mean intake. Linear regression for association of food group intakes with degree of fibrosis. * *p* < 0.05 adjusted for age, gender and BMI.

## Data Availability

The data that support the findings of this study are available from the corresponding author (G.P.A.) upon reasonable request.

## Supplementary Materials

The following are available online at https://www.mdpi.com/article/10.3390/nu14142808/s1, Figure S1: Participant Flow Chart. Table S1: Associations between food groups and clinical outcomes amongst individuals with NAFLD.
