# Supplementary material for: Development of Food Group Tree-Based Analysis and Its Association with Non-Alcoholic Fatty Liver Disease (NAFLD) and Co-Morbidities in a South Indian Population: A Large Case-Control Study
Source: Nutrients. 2022 Jul 8;14(14):2808. doi: 10.3390/nu14142808 (PMC9322963; doi:10.3390/nu14142808)
Supplement: Supplementary file 1 [file nutrients-14-02808-s001.zip › nutrients-1767921-supplementary.pdf]

Supplementary

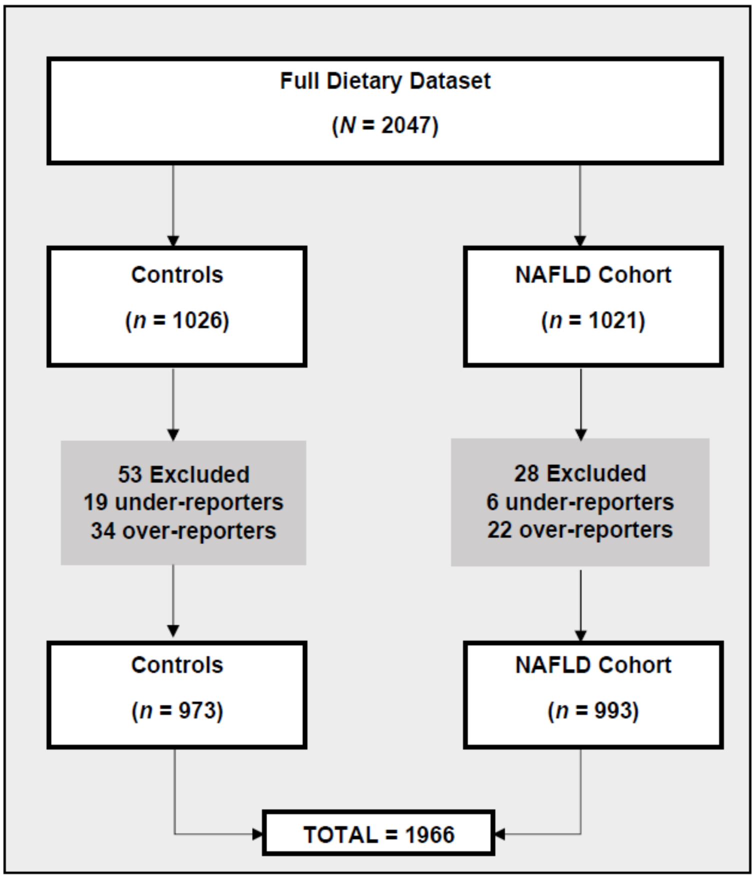

Figure S1. Participant Flow Chart.

Table S1: Associations between food groups and clinical outcomes amongst individuals with NAFLD.

	Diabetes Mellitus				Hypertension				Dyslipidaemia				CVD Events			
	***adj* OR**	**95% CI**		**P-value**	***adj* OR**	**95% CI**		**P-value**	***adj* OR**	**95%CI**		**P-value**	***adj* OR**	**95% CI**		P-value
**FOOD GROUP LEVEL-1**																
Cereals and Millets	0.999	0.997	1.001	0.152	0.999	0.997	1.001	0.402	1.001	0.999	1.004	0.311	0.997	0.992	1.001	0.182
Condiments and Spices	0.989	0.918	1.065	0.765	1.000	0.915	1.094	0.993	1.070	0.974	1.175	0.16	1.001	0.849	1.179	0.991
Fats and Edible Oils	1.002	0.973	1.033	0.873	1.055	1.012	1.099	0.013*	0.998	0.963	1.034	0.912	0.999	0.931	1.072	0.978
Fruits	0.997	0.967	1.028	0.847	0.949	0.911	0.989*	0.011*	1.001	0.966	1.039	0.94	1.004	0.934	1.078	0.922
Meat Fish Poultry	1.004	0.996	1.012	0.31	1.000	0.990	1.010	0.966	0.995	0.985	1.005	0.332	1.000	0.982	1.018	0.996
Milk and Milk Products	1.001	0.999	1.004	0.195	1.003	1.000	1.005	0.039	1.000	0.998	1.003	0.877	1.001	0.996	1.006	0.705
Nuts and Oil Seeds	0.996	0.983	1.008	0.50	1.005	0.991	1.020	0.499	0.995	0.980	1.010	0.493	0.971	0.942	1.001	0.061
Pulses and Legumes	1.003	0.992	1.014	0.634	1.003	0.990	1.016	0.657	0.990	0.976	1.003	0.139	1.005	0.979	1.032	0.702
Sugars	1.080	0.971	1.100	<0.001*	0.988	0.978	0.998	0.022*	0.988	0.977	0.999	0.04*	0.997	0.975	1.019	0.784
Vegetables	1.000	0.996	1.004	0.888	1.001	0.997	1.006	0.526	1.000	0.995	1.004	0.965	1.009	1.002	1.016	0.009*
**FOOD GROUP LEVEL-2**																
Refined Rice	0.998	0.996	1.00	0.032*	0.998	0.995	1.00	0.062	1.000	0.997	1.003	0.816	1.001	0.995	1.007	0.815
Refined Wheat	0.985	0.948	1.024	0.437	0.988	0.942	1.037	0.632	0.995	0.945	1.049	0.862	1.024	0.995	1.11	0.565
Wholegrain Rice	0.122	0.028	0.527	0.005*	0.456	0.102	2.030	0.303	0.521	0.419	0.891	0.027	0.124	0.110	0.210	0.436
Wholegrain Wheat	1.003	0.998	1.008	0.271	0.714	0.699	1.01	0.123	0.792	0.662	0.812	0.118	0.790	0.673	0.997	0.249
Wholegrains	1.032	0.918	1.160	0.599	0.979	0.842	1.136	0.776	0.960	0.808	1.141	0.644	0.990	0.973	1.007	0.125
Dried Condiments and Spices	1.005	0.890	1.136	0.933	0.975	0.841	1.129	0.733	1.123	0.942	1.338	0.197	0.224	0.190	0.390	0.493
Fresh Condiments and Spices	1.078	0.717	1.622	0.717	0.957	0.604	1.519	0.853	1.163	0.670	2.018	0.592	0.901	0.670	1.213	0.653
Animal Fats	2.144	1.300	3.536	0.003*	1.780	0.995	3.185	0.042	0.932	0.918	1.002	0.181	0.898	0.332		0.007*
Refined Plant Fat	1.000	0.954	1.049	0.99	0.946	0.884	1.013	0.111	0.998	0.937	1.064	0.957	0.791	0.285	2.195	0.951
Unrefined Plant Fat	0.961	0.847	1.091	0.542	1.01	0.878	1.161	0.894	1.083	0.919	1.277	0.342	2.455	1.280	4.710	0.246
Dried Fruits	0.999	0.866	1.153	0.991	0.989	0.827	1.161	0.88	1.063	0.089	1.264	0.475	1.004	0.872	1.157	0.456
Fresh Fruits	0.999	0.996	1.003	0.715	0.991	0.996	1.004	0.972	0.882	0.884	0.899	0.022	0.943	0.857	1.103	0.754
Eggs and Egg Products	0.995	0.963	1.029	0.788	1.003	0.966	1.041	0.874	0.997	0.952	1.045	0.904	0.964	0.983	1.041	0.228
Non-Oily Fish	1.051	0.973	1.135	0.205	0.981	0.883	1.089	0.718	1.011	0.878	0.878	1.163	0.941	0.739	1.199	0.794
Oily Fish	1.002	0.988	1.017	0.772	1.001	0.983	1.019	0.914	0.986	0.878	1.162	0.197	0.802	0.451	1.429	0.435
Shellfish	0.746	0.506	1.101	0.14	0.972	0.631	1.51	0.902	0.934	0.912	0.964	0.268	0.193	1.016	2.32	0.195
Red Meat	1.017	0.998	1.036	0.077	1.000	0.976	1.024	0.979	0.994	0.964	1.025	0.705	1.012	0.983	1.041	0.622
White Meat	0.990	0.968	1.013	0.388	1.013	0.989	1.038	0.295	0.976	0.941	1.012	0.186	1.008	0.937	1.084	0.833

Dried Milk and Milk Products	0.750	0.569	0.989	0.042	0.696	0.492	0.984	0.04*	1.143	0.803	1.628	0.458	1.758	0.663	4.657	0.257
Fresh Milk and Milk Products	1.001	0.999	1.003	0.453	1.002	0.999	1.004	0.236	1.002	0.999	1.005	0.213	1.004	0.998	1.01	0.234
Nuts	1.002	0.986	1.018	0.822	1.013	0.995	1.032	0.159	0.997	0.976	1.018	0.759	1.001	0.949	1.056	0.957
Oily Seeds	1.032	0.330	3.157	0.955	0.903	0.254	3.205	0.875	0.785	0.169	3.630	0.757	0.652	0.422	0.899	0.182
Dried Pulses and Legumes	0.678	0.560	0.892	0.807	0.713	0.688	0.988	0.691	0.673	0.619	0.690	0.02*	0.635	0.618	0.991	0.795
Fresh Pulses and Legumes	0.534	0.418	0.998	0.825	0.874	0.835	1.02	0.281	0.768	0.722	0.988	0.187	0.782	0.739	0.910	0.603
Refined Sugars	0.978	0.965	1.088	**<0.001***	0.993	0.981	1.004	0.2	0.988	0.922	1.016	0.068	0.989	0.962	1.016	0.412
Unrefined Sugars	0.712	0.702	0.903	0.833	0.931	0.798	1.087	0.368	0.797	0.606	1.048	0.104	0.379	0.151	0.951	0.039*
Leafy Vegetables	0.795	0.673	1.017	0.656	0.615	0.512	0.891	0.21	0.567	0.547	0.891	0.638	0.417	0.219	0.871	0.731
Other Vegetables	1.001	0.993	1.007	0.993	0.994	0.994	1.011	0.279	0.891	0.781	0.991	0.868	0.513	0.418	0.671	0.114
Roots and Tubers	0.885	0.816	0.984	0.415	0.894	0.883	1.010	0.584	0.810	0.784	1.012	0.77	0.819	0.715	1.021	0.554
**FOOD GROUP LEVEL-3**																
Baked	1.017	0.995	1.039	0.123	1.008	0.983	1.035	0.522	0.972	0.935	1.011	0.155	1.016	0.969	1.059	0.505
Boiled	0.799	0.698	1.00	0.168	0.771	0.767	0.810	0.035*	0.701	0.610	0.991	0.136	0.501	0.492	0.889	0.744
Fried	1.020	0.996	1.089	0.993	0.999	0.997	1.004	0.734	0.999	0.994	1.005	0.838	1.004	0.995	1.012	0.371
Roasted	0.997	0.993	1.001	0.113	0.996	0.991	1.001	0.119	0.995	0.990	1.001	0.096	1.001	0.990	1.012	0.86
Sauteed	1.000	0.999	1.001	0.647	1.001	0.999	1.002	0.596	1.000	0.999	1,002	0.643	0.998	0.995	1.002	0.394
Steamed	0.781	0.691	0.981	0.42	0.662	0.597	1.006	0.423	0.606	0.589	0.610	0.03*	0.710	0.690	0.918	0.907
Juice	0.986	0.965	1.001	0.208	1.003	0.977	1.029	0.825	0.983	0.948	1.019	0.351	1.023	0.977	1.071	0.327
Uncooked	0.627	0.597	0.997	0.261	0.711	0.700	0.819	0.239	0.593	0.491	0.991	0.827	0.661	0.513	0.891	0.664

*$p < 0.05$ adjusted for age, gender and BMI
